# Supplementary figures and images for: Evaluating antibody mediated opsonophagocytosis of bacteria via lab protocol: RAW 264.7 cell phagocytosis assay
Source: PLoS One. 2025 Sep 9;20(9):e0331445. doi: 10.1371/journal.pone.0331445 (PMC12419644; doi:10.1371/journal.pone.0331445)

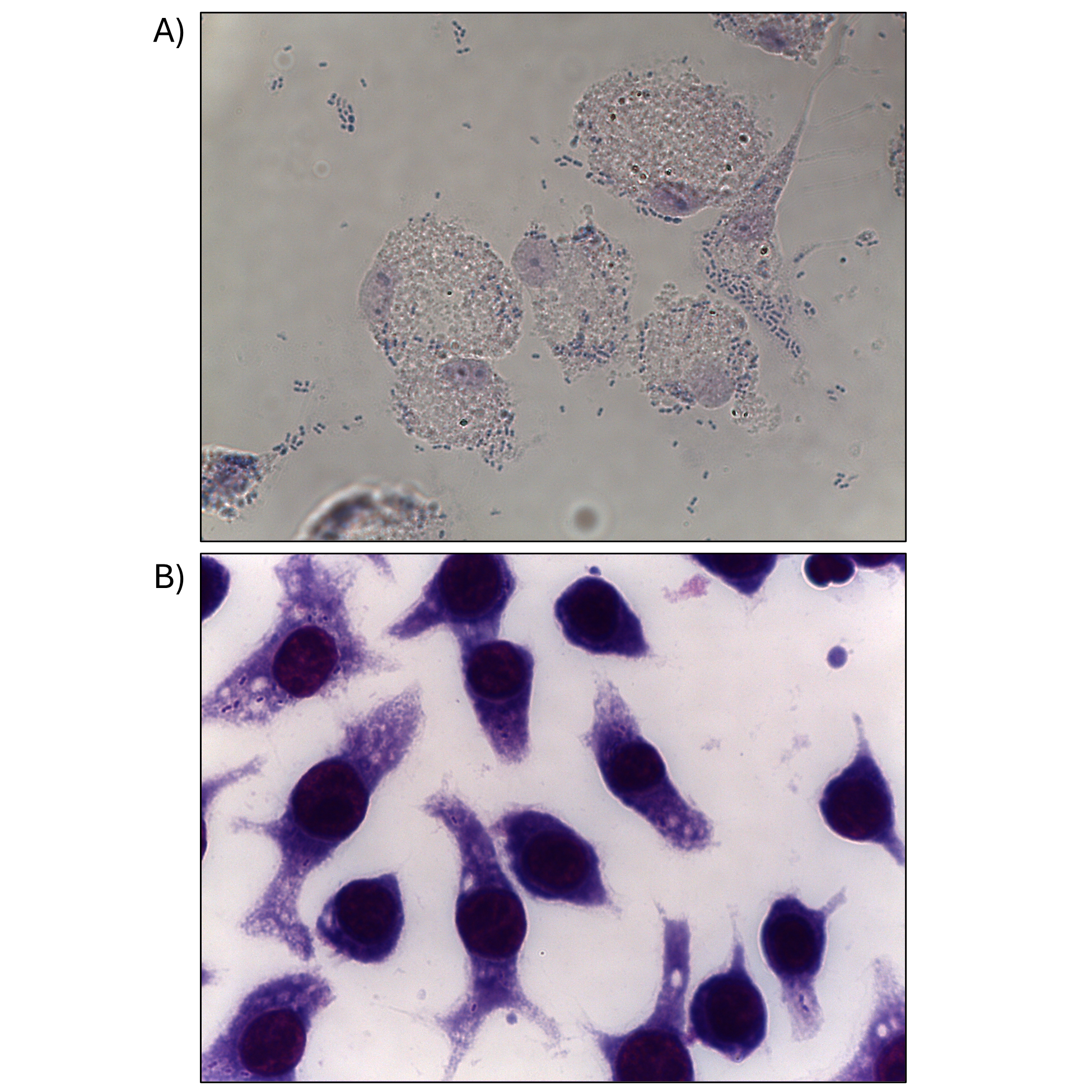

Supplement: S1 Fig — RAW 246.7 cells were exposed to A. baumannii with a multiplicity of infection of 1:20. Phagocytosis, washing, and fixing steps were performed as according to the protocol, but staining was not. (A) Cells were stained for only 10 seconds per stain, as opposed to the appropriate 1 minute for HEMA stain I and 45 seconds for HEMA stain II as listed in the protocol. (B) Cells were stained for one minute and 30 seconds per stain as opposed to the appropriate times listed in the protocol and above. These assays are unreadable. (TIF) [file pone.0331445.s002.tif]
