# Supplementary material for: Evaluating antibody mediated opsonophagocytosis of bacteria via lab protocol: RAW 264.7 cell phagocytosis assay
Source: PLoS One. 2025 Sep 9;20(9):e0331445. doi: 10.1371/journal.pone.0331445 (PMC12419644; doi:10.1371/journal.pone.0331445)
Supplement: S1 Protocol — The protocol described in this peer-reviewed article is published on protocols.io, (DOI: dx.doi.org/10.17504/protocols.io.yxmvmmr16v3p/v1). (PDF) [file pone.0331445.s001.pdf]

Aug 20, 2025

RAW 264.7 cell phagocytosis assay

DOI
dx.doi.org/10.17504/protocols.io.yxmvmmr16v3p/v1

Matthew Slarve[1], Travis Nielsen[1], Dongran Yu[1], Jordyn VanOrman[1], Brian Luna[1], Brad spellberg[1]
[1]University of Southern California

Matthew Slarve
University of Southern California

OPEN ACCESS

DOI: dx.doi.org/10.17504/protocols.io.yxmvmmr16v3p/v1

Protocol Citation: Matthew Slarve, Travis Nielsen, Dongran Yu, Jordyn VanOrman, Brian Luna, Brad spellberg 2025. RAW 264.7 cell phagocytosis assay. **protocols.io** **https://dx.doi.org/10.17504/protocols.io.yxmvmmr16v3p/v1**

License: This is an open access protocol distributed under the terms of the **Creative Commons Attribution License**, which permits unrestricted use, distribution, and reproduction in any medium, provided the original author and source are credited

Protocol status: Working
We use this protocol and it's working

Created: June 05, 2025

Last Modified: August 20, 2025

Protocol Integer ID: 219884

Keywords: Phagocytosis-killing assay, Macrophages , Lysing, evaluating phagocytosi, photographic phagocytosis assay, cell phagocytosi, phagocytosi, killing of phagocytosed microbial target, phagocytosed microbial target, macrophage, kill assay, vitro method, visible via 100x microscopy, cell, 100x microscopy

Funders Acknowledgements:
NIAID
Grant ID: 2R01 AI130060

Abstract

This protocol describes *in vitro* methods for evaluating phagocytosis using the macrophage-like cell line, RAW 264.7. The two methods discussed here include (1) a photographic phagocytosis assay, which is compatible with any target that is visible via 100x microscopy, and measures total uptake of target material per macrophage, and (2) the phagocytosis-kill assay, which measures killing of phagocytosed microbial targets by the macrophages.

Guidelines

Recommended controls

Designing appropriate controls for these macrophage phagocytosis assays depends on the goal of the assay itself. In the example of an experiment evaluating a therapeutic antibody's ability to opsonize a bacterial target, an appropriate negative control would be an isotype control antibody (EX: Fisher Scientific, cat# MAB002), however no positive control would be possible. Our lab has also used phagocytosis assays to evaluate immunostimulatory compounds, and in such cases our negative controls are cells that are not given IFN-γ, and positive controls are given IFN-γ, with experimental groups given our test compounds instead.

Materials

Materials and Media:

A	B	C	D
**Item**	**Vendor**	**Catalog number**	**Description**
RAW 264.7 cells	ATCC	TIB-71	Phagocyte cell line used for in vitro phagocytosis assays
DMEM cell media	Thermofisher	11965126	Supplement with 10% FBS to prepare complete media
Murine IFN-γ	Peprotech	315-05	Used to activate RAW 264.7 cells
Phosphate Buffered Saline (PBS)	VWR	45000-436	General purpose saline buffer
Hanks Buffered Salt Solution (HBSS)	VWR	45001-101	Supplemented buffer for washing and maintaining cells
Circular glass coverslips	VWR	48380-046	18 mm circular coverslips that fit into a 12 well plate
Vectamount	Vector laboratories	H-5501-60	A wet mount solution that mounts coverslips to microscope slides
HEMA I	VWR	10143-236	A stain used for visualizing phagocytosis
HEMA I I	VWR	10143-238	A stain used for visualizing phagocytosis
Triton-X-100	VWR	AAA16046-AE	Dilute to 0.1% in PBS to make a lysis buffer
CD-1 Mouse serum	Innovative Research, Inc.	IGMSCD1SER100ML	Serum for supporting cells during the phagocytosis process
Light microscope and mounted camera	Leica	ICC50 HD	For the microscopy-based assay, a high-quality mounted camera is recommended

RAW 264.7 Cells **ATCC Catalog #**TIB-71

DMEM, high glucose **Thermo Fisher Catalog #**11965126

Mouse IgG Isotype Control, FITC **peprotech Catalog #**31505

Dulbeccos phosphate-buffered saline (DPBS) **VWR International (Avantor) Catalog #**45000-436

HBSS (Hank's Balanced Salt Solution) 1X with Calcium and Magnesium, without Phenol red **VWR International (Avantor) Catalog #**45001-101

Cover glasses, round **VWR International (Avantor) Catalog #**48380-046

VectaMount® AQ Aqueous Mounting Medium **Vector Laboratories Catalog #**H-5501-60

Wright-Giemsa stain, VWR® Quick III™ Solution I for hematology **VWR International (Avantor) Catalog #**10143-236

Wright-Giemsa stain, VWR® Quick III™ Solution II for hematology **VWR International (Avantor)** **Catalog #**10143-238

TRITON™ X-100 (Polyethylene glycol tert-octylphenyl ether) **VWR International (Avantor)** **Catalog #**AAA16046-AE

Basic cell culture

5m

1 Remove a tube of RAW 264.7 cells from liquid nitrogen storage and thaw the cells by holding it in a 37 °C water bath, making sure not to let the water enter the cap.

2 Transfer the thawed cells into a 50 mL tube containing 9 mL complete media and remove the lingering DMSO by centrifuging at 300 x g, 00:05:00 and replacing the supernatant with 20 mL fresh complete media (DMEM + 10% FBS). 5m

3 Move the full 20 mL volume into a T75 flask (tissue culture treatment is not necessary) and disperse the cells evenly by gently sliding the flask back and forth as well as side to side 3-5 times each. Do not move the flask in a rotational pattern, or the cells will not disperse evenly. Incubate the cells under standard conditions of 37 °C with 5% CO_2. All further incubations should be done at these standard conditions unless otherwise stated.

4 When cells have formed a monolayer with 80-90% confluency (48:00:00 - 72:00:00 of growth), they are ready for passaging. Resuspend the cells by firmly slapping the side of the flask. Cells can be counted via trypan blue exclusion and should be seeded at 1e5 viable cell/mL. RAW 264.7 cells should be discarded after 15 passages.

Assay plate preparation (both photographic and killing assays)

8h

5 Using sterile forceps, aseptically transfer autoclaved circular glass coverslips into the bottom of a 12-well plate. Gently shake the plate to ensure that coverslips are flush with the floor of the well.

Note

This step is not required for a phagocytosis-killing assay.

6 Add 1 mL of RAW 264.7 cells that have been adjusted to 5e5 cell/mL, and gently slide the plate back and forth and side to side to evenly distribute the cells along the

coverslip. It is best to use RAW 264.7 cells that were collected at approximately 90% confluency.

7 If desired, stimulate the cells to differentiate into macrophages with the addition of 1 µg murine IFN-γ. Unstimulated cells will have reduced phagocytosis activity.

8 Incubate the cells under standard conditions Overnight . Observe the cells via inverted microscope the next day to confirm that a monolayer has formed with ~90% confluency, and macrophage-like morphology (if IFN- γ activated).

8h

Infection (both photographic and killing assays)

1h 5m

9 Aspirate the media from each well, being careful not to disturb the adhered cells.

10 Wash each well once by slowly adding 1 mL HBSS that has been pre-warmed to 37 °C , and aspirating. Ensure that the HBSS is added to the wall of the well, not directly onto the cells.

11 Add pre-warmed HBSS supplemented with 10% CD-1 mouse serum (Innovative Research Inc #IGMSCD1SER100ML) any experimental agents (drugs, antibodies, etc.) that are being investigated, and the inoculum being studied (bacteria, yeast, polystyrene beads, etc.) in a final volume of 1 mL . An example for an experiment investigating the effect of a monoclonal antibody on phagocytosis of bacteria is as follows:

- 800 µL HBSS
- 100 µL CD-1 mouse serum
- 50 µL antibody suspension
- 50 µL bacterial suspension (2e8 CFU/mL)

Note

We have found that using 50 uL of 2e8 CFU/mL gives a multiplicity of infection (MOI) of 20 bacterial cells per one macrophage has been ideal in our model, but may differ for other experimental designs.

12 Gently shake the plate back and forth and side to side to distribute the bacteria evenly in the well. Then centrifuge the plate at 300 x g, 00:05:00 with slow deceleration to bring the inoculum to the floor of the plate where macrophages can access it efficiently.

5m

13 Incubate the plate at 37 °C for 01:00:00 to allow phagocytosis to occur. We have found that 5% CO_2 is not critical for this step.

1h

14 **Optional for bacterial phagocytosis assays:** After one hour of phagocytosis, add gentamicin to a final concentration of 200 µL , and incubate for an additional hour to kill any bacteria that have not been phagocytosed. This step prevents non-phagocytosed bacteria from confounding the colony forming unit count in killing assays, and may reduce background in photographic assays.

15 Wash the cells 3x with 1 mL of warm HBSS. For the first wash, use a p1000 pipettor to expel the HBSS at medium pressure directly onto the cells to dislodge the remaining inoculum (bacteria, yeast, polystyrene beads, etc.) that may be adhered to the outside of the cell, or the floor of the well. For the second and third wash, dispense the HBSS onto the side of the well, and let it flow down over the cells. Continued direct washing onto the cells may cause them to detach.

> Note
>
> For photographic phagocytosis assays, proceed to the fixing, staining, and visualizing steps. For killing assays, proceed to the lysing and plating steps.

Fixing, staining, and visualizing (photographic assay only)

8h 1m 45s

16 After cells have been thoroughly washed, tilt the 12-well plate and slowly dispense 1 mL of methanol onto the wall of the well, allowing the methanol to slowly cover the cells. If this step is done too quickly, the methanol will strip the cells from the coverslip, so proceed cautiously. Incubate for at least one minute to fix the cells to the coverslip.

Note

The timing for the following staining steps is critical, and too much exposure (particularly to HEMA II) will ruin the photo quality. Furthermore, we recommend only actively staining 4 wells at a time, as trying to stain too many wells at once can greatly increase the variability of stain quality between wells. Any wells that are not being actively stained should be filled with 1 mL PBS to prevent cells from drying and preserve morphology.

17 Aspirate the methanol and add 1 mL HEMA I stain. Incubate for 00:01:00 , then promptly aspirate the stain and add HEMA II. Incubate with HEMA II for 00:00:45 , quickly aspirate, and wash 3x with DI water. After the third wash is aspirated, add 1 mL DI water. Leave the cells submerged until all staining is done, and it is time to mount.

1m 45s

18 Prepare a hook for lifting slides by taking a needle (25 gauge is appropriate) and bending the beveled end against a solid surface.

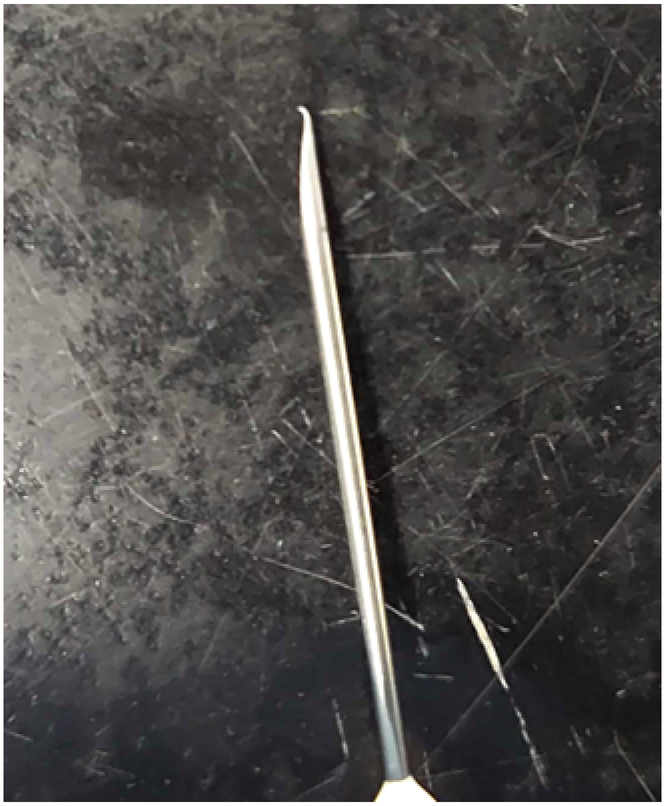

Figure 1: Photograph of a slide hook

19 Place a dot of vectamount approximately half the diameter of the coverslip on a clean microscope slide.

20 Slide the bent bevel underneath a coverslip in the well (ensure that the coverslip is submerged in DI water) and firmly tilt the slide upwards. Grab it with forceps and place it cell-side down onto the droplet of vectamount. Gently press down with forceps to squeeze out any air bubbles that may have formed.

21 Allow the slides to dry on the bench Overnight . 8h

Note

We have found it useful to blind the following step by using generic labels on the microscope slides (group 1, group 2, etc.) and having a lab worker who does not know which treatments correspond to which labels take the photos. This can reduce the bias that may occur in the photography step.

22 Using oil immersion microscopy and a microscope camera, take 5 separate, non-overlapping photos of each coverslip, making sure that the visible fields are representative of the character of the coverslip as a whole.

23 Measure the phagocytosis by counting the particles (individual bacterial or yeast cells, polystyrene beads, etc.) that are visible inside the macrophages using an image processing software such as Fiji's cell counting tool (https://imagej.net/software/fiji/). Macrophages that are not fully visible in the frame of the image should not be counted.

Note

We have found that when assessing bacterial phagocytosis, it may be useful to incubate the cells with 200 ug/mL gentamicin for one hour at 37C prior to the following lysis step to eliminate bacteria that are stuck to the wells but have not been phagocytosed.

Lysing and plating (killing assay only) 8h 2m

24 Lyse the cells by adding 1 mL 0.1% Triton-X-100 to the well and gently pipetting up and down. The cells should be visibly removed from the bottom of the well, and the

Triton-X-100 suspension should become cloudy with lysate. Microbial cells will remain viable.

25 Collect the suspensions from each well, transfer them into sterile microfuge tubes, and centrifuge at 4000 x g, 00:02:00 to pellet the microbes. Discard the supernatant to remove the bulk of Triton-X-100 which may hinder microbial growth. 2m

26 Prepare serial dilutions in PBS, and plate on media that is suitable for the microbe being tested (Ex: Tryptic soy agar for most bacteria). Incubate Overnight , and count colonies on the following day to assess the number of viable microbes. 8h
