# Supplementary material for: Evaluating antibody mediated opsonophagocytosis of bacteria via lab protocol: RAW 264.7 cell phagocytosis assay
Source: PLoS One. 2025 Sep 9;20(9):e0331445. doi: 10.1371/journal.pone.0331445 (PMC12419644; doi:10.1371/journal.pone.0331445)
Supplement: Attachment — Submitted filename: Dear Author.docx [file pone.0331445.s003.docx]

Dear Author,

This manuscript is potentially acceptable pending the resolution of major issues outlined below. Your study on measuring phagocytosis by RAW 246.7 cells and evaluating the effect of monoclonal antibodies on Acinetobacter baumannii addresses an important and practical topic in immunology and pharmacology. We appreciate your effort in this valuable research. However, we suggest the following points to improve your manuscript:

1. Please provide more detailed explanations of the phagocytosis mechanism and its biological stages to help readers better understand the underlying processes.
2. Include information about experimental controls and the reproducibility of your results in the methods section to enhance the scientific rigor of your work.
3. Discuss the limitations and potential challenges of the methods you introduced to make the manuscript more comprehensive and transparent.
4. Consider expanding the discussion to include the applicability of these methods to other microorganisms or at least mention this aspect to broaden the scope of your research.
5. Review and refine the sentence structure and writing style of the abstract to make it clearer, more fluent, and more suitable for a specialized audience.
6. Figure 1 does not have sufficient transparency and clarity; please improve its quality.
7. The full title is identical to the short title and should be corrected to better reflect the content.
8. The description of experimental procedures lacks sufficient detail. Please provide comprehensive information on cell culture conditions, bacterial concentrations, incubation times, and sample preparation steps.
9. It is important to include descriptions of both negative and positive controls to validate your results and ensure the reliability of the assays.
10. The criteria and quantitative measures used for assessing phagocytosis and bacterial killing in both the imaging and killing assays need to be clearly defined.
11. Please specify environmental conditions such as temperature and pH, as well as details about the equipment used (e.g., microscope type, colony counting devices) to facilitate accurate replication.
12. Discuss any limitations or potential challenges associated with the methods to provide a balanced perspective.
13. Indicate the number of independent experimental replicates performed to support the statistical validity of your findings.
14. Addressing these points will significantly strengthen the methods section and improve the overall quality of your manuscript.

We hope that by addressing these points, your manuscript will be well-prepared for publication in PLOS ONE. We look forward to reviewing your revised manuscript and wish you success.

Best regards
