# Supplementary material for: Evaluating antibody mediated opsonophagocytosis of bacteria via lab protocol: RAW 264.7 cell phagocytosis assay
Source: PLoS One. 2025 Sep 9;20(9):e0331445. doi: 10.1371/journal.pone.0331445 (PMC12419644; doi:10.1371/journal.pone.0331445)
Supplement: Attachment — Submitted filename: Response to reviewers.docx [file pone.0331445.s004.docx]

**Dear Reviewers,**

**Thank you for your constructive feedback on our manuscript and protocol for this lab protocol submission to PLOSone. We feel that your comments have been excellent in improving the quality of this submission, and we hope that the changes we have made to address your concerns have been satisfactory. Below, please find a point-by-point breakdown of our response to each of the comments left by both reviewers. Thank you for your contribution.**

1. **Please provide more detailed explanations of the phagocytosis mechanism and its biological stages to help readers better understand the underlying processes.**

- A brief introduction to the fundamentals of phagocytosis has been added, including citations of papers with detailed descriptions of the process for interested readers.

2. **Include information about experimental controls and the reproducibility of your results in the methods section to enhance the scientific rigor of your work.**

- These sections have been added to the methods section of the main manuscript

3. **Discuss the limitations and potential challenges of the methods you introduced to make the manuscript more comprehensive and transparent.**

- Limitations and potential challenges have been added to the conclusion

4. **Consider expanding the discussion to include the applicability of these methods to other microorganisms or at least mention this aspect to broaden the scope of your research.**

- A statement has been added to the conclusion that indicates that this assay works for other microbes.

5. **Review and refine the sentence structure and writing style of the abstract to make it clearer, more fluent, and more suitable for a specialized audience.**

- The abstract has been thusly adjusted

6. **Figure 1 does not have sufficient transparency and clarity; please improve its quality.**

- The older figure has been replaced with a higher resolution image.

7. **The full title is identical to the short title and should be corrected to better reflect the content.**

- The titles have been updated and are now different.

8. **The description of experimental procedures lacks sufficient detail. Please provide comprehensive information on cell culture conditions, bacterial concentrations, incubation times, and sample preparation steps.**

- The protocol has been updated to clearly specify the culture and incubation conditions, concentration of the bacterial inoculum, and all preparation of samples.

9. **It is important to include descriptions of both negative and positive controls to validate your results and ensure the reliability of the assays.**

- A description of negative and positive controls has been added to the methods section of the manuscript, and as an addendum to the end of the protocol.

10. **The criteria and quantitative measures used for assessing phagocytosis and bacterial killing in both the imaging and killing assays need to be clearly defined.**

-These descriptions are now included in both the methods and expected results sections of the main manuscript.

11. **Please specify environmental conditions such as temperature and pH, as well as details about the equipment used (e.g., microscope type, colony counting devices) to facilitate accurate replication.**

- The protocol has been updated to specify the culture conditions, as well as the microscope and camera that our lab uses.

12. **Discuss any limitations or potential challenges associated with the methods to provide a balanced perspective.**

- This has been added to the conclusion section of the manuscript

13. **Indicate the number of independent experimental replicates performed to support the statistical validity of your findings.**

- A section has been added to the methods section that addresses this concern

**Reviewer #2: -I recommend revising the title to specify the lab protocol in title.**

-The title has been thusly modified.

**-Add methodology and results to Abstract. Compare the results obtained from two used method.**

**-** The abstract has been enhanced with a brief description of the methodology, and explanation of how our methods differ from previous protocols.

**-Introduction is so brief. Explain more about currently available protocols, introduce the novel monoclonal antibodies (their characteristics, advantages, …).**

**-** The introduction has been enhanced with additional information about the other available protocols, and some context about the novel MAbs.

**-List the MABs in materials (table) in protocol.**

**-** Because this protocol is meant to cover more than just monoclonal antibody investigations, we instead updated the table to include any experimental substance being used by the investigator.

**-In protocol, mention the CFU/mL of 50 uL bacterial suspension.**

- the protocol has been updated to fix this oversight. **-It is recommended to add figure for results represented in lines 46-50.**

- Examples of improperly stained figures have been added as supplemental figure S1. These new supplementary figures have also been referenced in the text at the aforementioned lines.
